# High Expression of DEPDC1 Promotes Malignant Phenotypes of Breast Cancer Cells and Predicts Poor Prognosis in Patients With Breast Cancer

**DOI:** 10.3389/fonc.2019.00262

**Published:** 2019-04-12

**Authors:** Huishan Zhao, Mingwei Yu, Laijian Sui, Benjiao Gong, Bo Zhou, Jian Chen, Zhaohua Gong, Cuifang Hao

**Affiliations:** ^1^Reproductive Medicine Centre, The Affiliated Yantai Yuhuangding Hospital of Qingdao University, Yantai, China; ^2^Department of Orthopedics, The Affiliated Yantai Yuhuangding Hospital of Qingdao University, Yantai, China; ^3^Central Laboratory, The Affiliated Yantai Yuhuangding Hospital of Qingdao University, Yantai, China; ^4^Department of Special Education, Binzhou Medical University, Yantai, China; ^5^Department of Oncology, The Affiliated Yantai Yuhuangding Hospital of Qingdao University, Yantai, China

**Keywords:** breast cancer, DEPDC1, TCGA, proliferation, PI3K/AKT/mTOR, GSEA

## Abstract

DEP domain containing 1 (DEPDC1) is a novel tumor-associated gene, which is aberrantly expressed in multiple types of cancer and involves in tumorigenesis and cancer progression. Here, we examined the functional involvement and underlying mechanism of DEPDC1 in breast cancer. In this study, the immunohistochemistry results demonstrated that DEPDC1 was high-expressed in breast cancer tissues compared with the paired adjacent normal breast tissues, and its tendency at protein level was consistent with mRNA level from TCGA data. Moreover, DEPDC1 mRNA level revealed the strongest association with poor prognosis and development in breast cancer. *In vitro* assays showed that DEPDC1 overexpression resulted in significant promotion of proliferation by regulating cell cycle in MCF-7 cells, whilst an opposite effect was found in the MDA-MB-231 cells with DEPDC1 deletion. Notably, further investigation indicated DEPDC1's ability of promoting breast cancer cells migration and invasion. In addition, we discovered that DEPDC1 caused hyper-activation of PI3K/AKT/mTOR signaling in breast cancer cells. Therefore, the increased DEPDC1 expression in breast cancer is correlated with disease progression and poor survival, which suggested that DEPDC1 might be a potential therapeutic target against this disease.

## Introduction

Breast cancer is the most common carcinoma and estimated to have the highest cancer incidence rate among woman in the world (1). Radical mastectomy and chemotherapy are effective for ~85–90% patients with localized breast cancer, with a 5-years overall survival (2). However, over 30% of patients with breast carcinoma develop local invasion and distant metastasis during progression of their disease, leading to high mortality rate (3, 4). Unfortunately, The 5-years overall survival rate of breast cancer patients with metastasis is only ~10–18%, what's worse, incidence rate of breast cancer in young women is increasing and frequently metastatic (5, 6). Therefore, understanding the potential mechanisms associated with local invasion and distant metastasis has a critical significance in formulating treatment and early diagnosis.

DEP domain containing 1 (DEPDC1) is a recently identified novel oncogene and highly conserved from Caenorhabditis elegans to mammals (7–9). In human, DEPDC1 gene is located at 1p31.3, and the DEP (Disheveled, EGL-10, Pleckstrin) domain exists in the N-terminal regions (7). Two DEPDC1 transcriptional variants were found by northern blot and 5′-RACE analysis, and both of them were highly expressed in bladder cancer cells (8). Detailed sequence analysis showed that DEPDC1-V1 variant contained 12 exons encoding 811 amino acid residues whereas DEPDC1-V2 variant lacked one in-frame exon with 527 amino acid residues (8).

DEPDC1 was firstly reported in bladder cancer with aberrantly high expression; it acted as a transcriptional repressor by forming a complex with zinc finger protein 224 (ZNF224) to suppress A20 transcription, leading to the activation of anti-apoptotic pathway through activating NF-κB pathway (10). In recent years, studies showed that DEPDC1 was also aberrantly overexpressed in lung cancer, breast cancer, hepatocellular carcinomas and prostate cancer, and had prognostic value for predicting outcomes in patients with bladder cancer and lung cancer (9, 11–15). Moreover, its roles in promoting tumor development were gradually discovered. DEPDC1 was predominantly expressed during cell interphase and necessary for the proper division in metaphase, as a result of its silence leading to a significant mitotic arrest (16). In addition, DEPDC1 regulated microtubule-targeting chemotherapeutics to induce apoptosis through enhancing JNK-dependent degradation of the BCL-2 family protein MCL1 (17). Notably, one study also demonstrated that knockdown DEPDC1 resulted in significant inhibition of migration, proliferation and delay in cell cycle progression in nasopharyngeal cancer cells (18).

Previously studies had indicated that DEPDC1 may act as an early molecular marker for breast cancer (9, 19, 20). However, the biological function and mechanism of DEPDC1 in breast cancer remain elusive. In this study, we would investigate the clinical significance and functional implication of DEPDC1 in breast cancer. We confirmed that DEPDC1 was highly expressed in breast cancer tissues in comparison to normal tissues (2 cm adjacent from the tumor), and deletion of DEPDC1 reduced growth, migration and invasion in breast cancer cells. Moreover, to explore the mechanism of DEPDC1 correlated with cell proliferation and poor prognosis of breast cancer, we carried out gene set enrichment analysis (GSEA) for target genes of DEPDC1 in breast cancer patients. We predicted that DEPDC1 could activate the signaling of PI3K/AKT/mTOR in breast cancer cells. Further, we validated the prognostic significance of DEPDC1 by using multiple approaches and independent sets.

## Materials and Methods

### Cell Lines and Culture Conditions

Human breast cancer cells MCF-7 and MDA-MB-231 were purchased from American Type Culture Collection (ATCC, Manassas, VA, USA). They were incubated in RPMI-1640 medium supplemented with 1% penicillin/streptomycin and 10% fetal bovine serum (FBS, Gibco, Thermo Fisher Scientific, Waltham, MA, USA), at 37°C with an atmosphere of 5% CO_2_ and 95% humidity.

### Plasmids and Transfection

DEPDC1-flag and control vector plasmids were kind gifts from Professor Toyomasa Katagiri (The University of Tokyo, Tokyo, Japan). The siRNAs against DEPDC1 and control siRNA were chemically synthesized by Shanghai GeneChem Co., Ltd. (Shanghai, China). 1 μg of DEPDC1-flag or control vector was transfected into MCF-7, respectively, using the X-tremeGENE HP DNA Transfection Reagent (Roche Diagnostics GmbH, Mannheim, Germany) according to the manufacturer's instructions. MCF-7 cells with stably expressed DEPDC1-flag or vector-flag were selected with G418 (Calbiochem, San Diego, CA, USA). MDA-MB-231 Cells were transfected with DEPDC1-siRNA or corresponding control vector, respectively, for relevant RNA interference experiments. The siRNAs sequences were listed in Supplementary Table 1.

### RNA Isolation and Reverse Transcription-Polymerase Chain Reaction (RT-PCR)

Following the manufacturer's protocol, total RNA was isolated from MCF-7 and MDA-MB-231 cells using Trizol reagent (Invitrogen, Carlsbad, CA). cDNA was generated from each 1 μg RNA sample using QuantiTect Reverse Transcription Kit (Qiagen GmbH, Hilden, Germany). PCR amplifications were performed using Ex Taq hot start version (Takara Bio Inc., Otsu, Japan) following the manufacturer's instructions. The PCR products were separated on 2% agarose gel (NuSieve; FMC, Rockland, ME, USA) and visualized by Syber Green.

### Western Blot Analysis and Antibodies

Whole cell lysates were separated with 10% SDS-PAGE and transferred to nitrocellulose membrane (Millipore, MA). Following being blocked with 5% skimmed milk to block non-specific proteins for 1 h at room temperature, membranes were treated with corresponding primary antibody overnight at 4°C, and then followed by peroxidase-conjugated anti-mouse or anti-rabbit secondary antibodies for 1 h at room temperature. Protein bands were visualized using the Tanon High-sig ECL (Tanon Science and Technology Co., Ltd., Shanghai, China) and analyzed with Vilber Fusion Fx5 Spectra (Vilber Lourmat, Marne La Vallée, France). The anti-DEPDC1 antibody was purchased from abcam Inc. (Abcam, Cambridge, MA, USA). GAPDH antibody was obtained from Santa Inc (Dallas, TX, USA). AKT antibody (Cell Signaling Technology, USA), p-AKT (Ser473) (Cell Signaling Technology, USA), PI3K antibody (Santa Cruz, USA), p-PI3K p85 (Tyr458) antibody (Santa Cruz, USA), mTOR antibody (Abcam, USA) and p-mTOR (Ser2448) antibody (Cell Signaling Technology, USA) were used to analyze AKT/PI3K/mTOR signal pathway. HRP-conjugated secondary antibodies were from ZSGB-BIO (Beijing, China).

### Immunohistochemical Staining of DEPDC1 in Breast Specimens

66-pair breast cancer and adjacent normal tissues were sectioned at a thickness of 3 μm. All tissues sections were dewaxed and rehydrated using routine methods, and then incubated with 3% H_2_O_2_ for 10 min in a wet box. After being blocked with 3% BSA, slides were immunostained with primary antibodies anti-DEPDC1 (Abcam, Cambridge, MA, USA) at a dilution of 1:100 in a humidified box at 4°C overnight. Following three times washing, sections were incubated in the secondary antibody anti-rabbit (1:400, OriGene Technologies, Inc., Beijing, China) for 30 min at room temperature. Then stained the slides with 3,3′-diaminobenzidine (DAB) and counterstained with haematoxylin following the manufacturer's protocol. Following routine dehydration, mounted the coverslips on glass slides. Immunohistochemical images were taken with Leica Camera.

### *In vitro* Cell Proliferation Assay

Cell suspensions were plated in 96-well plate (3,000 cells/200 μl/well) in quadruplicate and evaluated following a period of incubation (overnight, day 3, day 4, and day 5). After removing the medium, 100 μl 10% Cell Counting Kit-8 (Dojindo Molecular Technologies, Inc., Kumamoto, Japan) was added to each well and incubated for an additional 1 h at 37°C according to the manufacturer's instructions. Subsequently, cell viability was determined at the wavelength of 450 nm using a spectrophotometer (BioTek Instruments, Inc., Winooski, VT, USA).

### Flow Cytometry Assay

Cells in the log phase of growth were trypsinized, washed with PBS and fixed with 70% ice-cold ethanol in PBS overnight at 4°C. After washing three times with PBS, cells were treated with 50 μg/ml RNAase and 50 μg/ml propidium iodide in the dark for half an hour at room temperature. Data acquisition was analyzed using MoFlo XDP (Beckman Coulter, Inc.).

### *In vitro* Cell Migration and Invasion Assay

Cells (1 × 10^5^/600 μl/well) were seeded into 12-well plates and cultured overnight at 37°C to form a confluent monolayer. Scraped an artificial wound in the monolayer with a 200 μl pipette tip and washed three times with PBS. Traced and recorded the activity of cells every 6 h using an inverted microscope. The wound was analyzed by Image J software (version 1.62; National Institute of Health, Bethesda, MD, USA). The invasion assay using transwell were performed as described previously (21).

### Datasets Collection

The microarray data were downloaded from the Gene Expression Ominibus (GEO) public database under accession number GSE29044 and GSE109169, which were used for detecting the differential expression of DEPDC1 in cancer and normal tissues. Gene expression profiles of breast cancer were downloaded from The Cancer Genome Atlas (TCGA) database (http://cancergenome.nih.gov/) and corresponding clinical data from the cBioPortal for Cancer Genomics (available online: http://cbioportal.org).

### Gene Set Enrichment Analysis

We used Gene Set Enrichment Analysis (GSEA v2.0, available online: http://www.broad.mit.edu/gsea/) to analyze the association between expression of DEPDC1 and biological processes/pathway, phenotypes. Pre-defined gene set were obtained from the Molecular Signatures Database, MSigDB (http://software.broadinstitute.org/gsea/msigdb).

Gene sets: BENPORATH_PROLIFERATION, FISCHER_G2_M_CELL_CYCLE, POOLA_INVASIVE_BREAST_CANCER_UP, REACTOME_PI3K_AKT_ACTIVATION, METASTASIS_OF_BREAST_CANCER_ESR1_UP, HALLMARK_PI3K_AKT_mTOR_SIGNALING. Samples from the TCGA datasets were divided into high- or low-DEPDC1 expression groups using the median as the cutoff. Default settings were used and thresholds for significance were determined by permutation analysis (1000 permutations). False Discovery Rate (FDR) was calculated. A gene set is considered significantly enriched when the FDR score is < 0.25.

### Statistical Analysis

Data are presented as the mean ± standard deviation. The results of unpaired and paired samples were analyzed by independent and paired sample *t*-test, respectively. DEPDC1 levels from stage I/T1 to higher stage/T were performed using a two-sided Student's *t*-test for two-group comparisons and by one-way ANOVA, followed by a Bonferroni *post-hoc* test, for multiple group comparisons. The Kaplan–Meier (KM) curve was conducted to assess the association between the expression level of DEPDC1 and survival time of patients with breast cancer. All statistical analyses were performed with the GraphPad prism 5 (Graphpad Software, Inc., La Jolla, CA). Statistical significance was considered significant when *p*<*0.05*. ^*^p < 0.05, ^**^p < 0.01, ^*****^p < 0.0001.

## Results

### DEPDC1 Expression Is Upregulated in Human Breast Cancer

**Figure 1:**
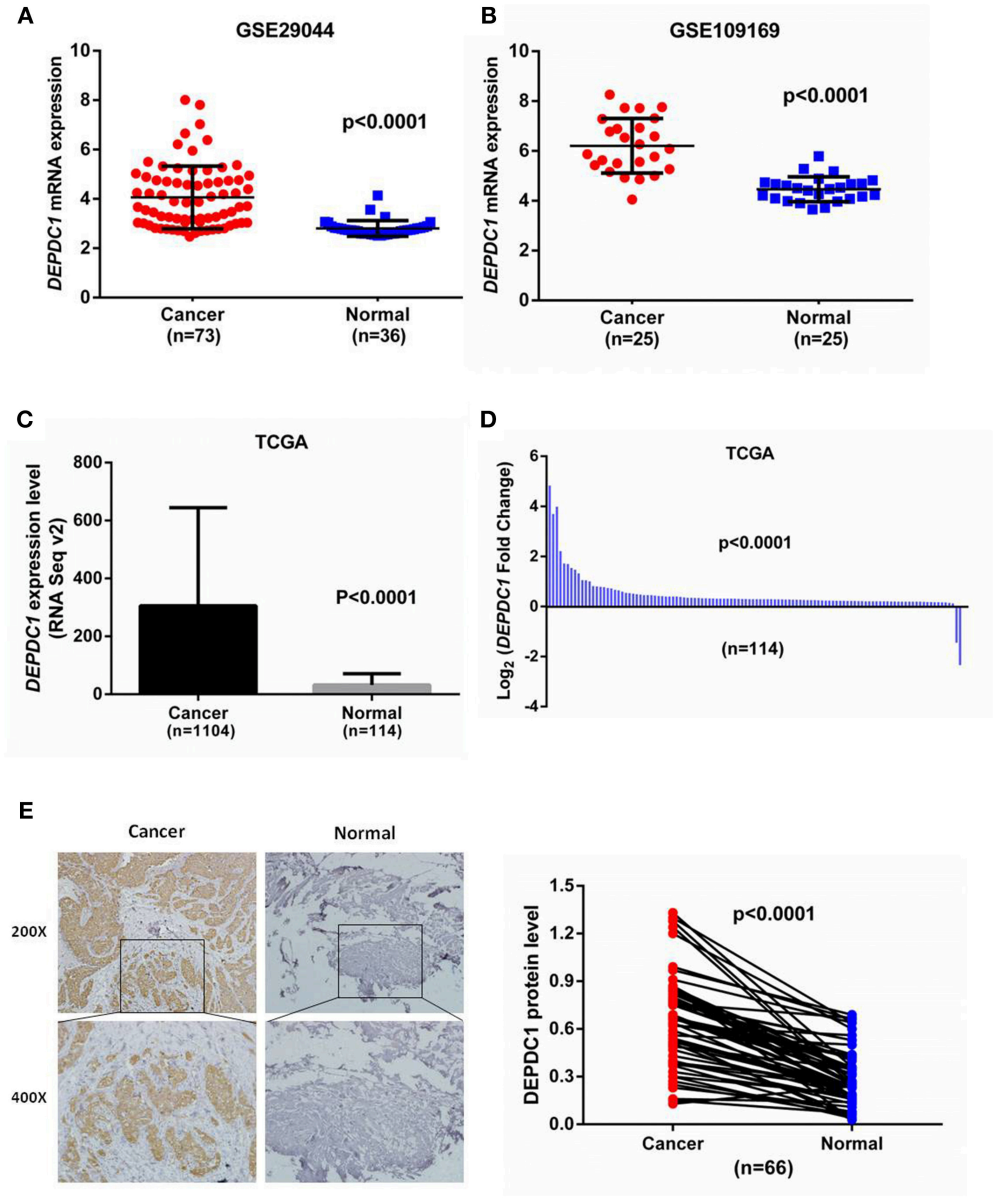
DEPDC1 was significantly upregulated in breast cancer. **(A)** DEPDC1 mRNA level was obviously higher in breast cancer specimens than in normal tissues by searching GEO dataset (GSE29044). **(B)** DEPDC1 expression was upregulated in human breast cancer compared to their paired normal tissues by searching GEO dataset (GSE109169). **(C,D)** The mRNA level of DEPDC1 was significantly increased in breast cancer tissues of the unpaired **(C)** and paired **(D)** via analyzing TCGA_BRCA database. **(E)** The DEPDC1 immunohistochemical staining images of 66 paired human breast cancer samples (left) and adjacent normal tissues (right).

To identify the role of DEPDC1 in breast cancer, we initially explored its expression profiles between breast cancer and normal breast tissues by analyzing microarray data from GEO database. The data from GSE29044 showed that DEPDC1 mRNA level was significantly higher in breast cancer tissues than in normal tissues (Figure 1A). GSE109169 is a publically available microarray database consisting of 25 paired breast cancer specimens, which demonstrated that DEPDC1 expression was upregulated in human breast cancer (Figure 1B). Further, we evaluated the mRNA level of DEPDC1 by searching TCGA_BRCA database. An increased transcript level of DEPDC1 was found in breast cancer compared with normal breast tissues (Figure 1C). To further verify the aberrant expression of DEPDC1, 114 pairs of breast cancer tissue samples from TCGA database were compared. DEPDC1 was significantly enhanced in tumor tissues compared with matched normal tissues (Figure 1D).

To further validate the protein levels of DEPDC1 in breast cancer, we examined the DEPDC1 expression level in breast cancer tissues which were collected from Yantai Yuhuangding Hospital using immunohistochemistry. Similar to the mRNA microarray results, a raised DEPDC1 protein level was discovered in breast cancer tissues (Figure 1E). Hence, these findings indicated that DEPDC1 might associate with the tumorigenesis of breast cancer.

### Upregulated DEPDC1 Is Associated With Advanced Stage, Metastasis, and Malignant Degree of Breast Cancer

**Figure 2:**
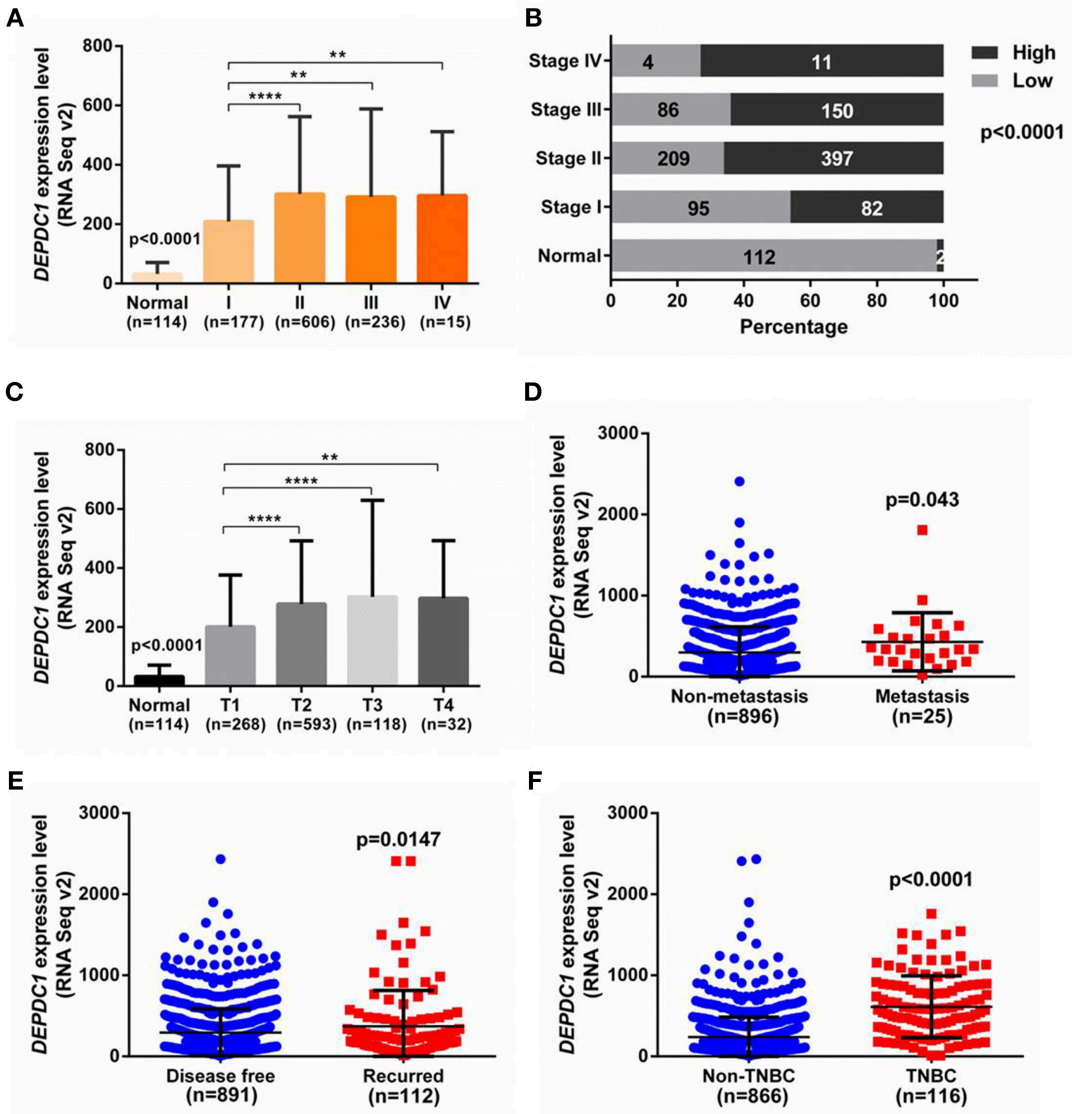
The association of DEPDC1with advanced stage, metastasis and malignant degree of breast cancer was analyzed from TCGA_BRCA dataset. **(A)** Correlation analysis was performed between expression level of DEPDC1 and clinical AJCC stages. DEPDC1 was correspondingly increased with the increase of clinical AJCC stage of breast cancer. **(B)** Constituent ratio with high level of DEPDC1 was drastically different between normal samples and I, II, III, or IV stage of breast cancer tissues. **(C)** DEPDC1expression level was increased as breast cancer tumor size (T) growth and distant metastatic relapse status **(D)** from TCGA_BRCA data. **(E)** Scatter plot of relative DEPDC1 mRNA level in breast cancer patients with and without recurrence was compared. **(F)** The expression of DEPDC1 was higher in TNBC than in non-TNBC of breast cancer. *p < 0.05, **p < 0.01 and ****p < 0.0001.

To investigate the role of DEPDC1 in breast cancer progression, we analyzed the expression level of DEPDC1 in different stages of breast cancer patients from TCGA. As shown in Figure 2A, DEPDC1 expression level was significantly enhanced in tumor stages compared with normal breast tissues. And with the development of breast cancer malignancy, the expression level of DEPDC1 was increased accordingly compared primary tumor stage. To further observe the role of DPEDC1 expression in breast cancer progression, constituent ratio analysis was performed in 1,034 primary breast cancer tissues and 114 normal samples with the median as the cutoff. In normal breast tissues, only 2/114 (2%) cases exhibited high expression level for DEPDC1 (>median). However, in stage I, II, III, and IV stage of breast cancer tissues, 82/177 (46%), 397/606 (66%), 150/236 (64%), and 11/15 (73%) of cases showed high expression level for DEPDC1, respectively. The percentage with high level of DEPDC1 was drastically different between normal samples and breast cancer stage I, II, III, or IV tissues (p < 0.0001), suggesting that DEPDC1 was associated with breast cancer progression and might have prognostic significance for breast cancer patients (Figure 2B). According to staging criteria from the American Joint Committee on Cancer (AJCC), T1-4 is defined from primary tumor and closely related to tumor size and cell proliferation. DEPDC1 expression level was significantly increased as T stage progressed compared with normal breast tissues (p < 0.0001, Figure 2C). Also significant difference of DEPDC1 expression was found among the different stages and primary tumors (Figure 2C). “Metastasis” represents the defined absence or presence of distant spread or metastases to locations via vascular channels or lymphatics beyond; “Disease free” represents disease free status since initial treatment. It also exhibited that DEPDC1expression was significantly associated with metastasis and recurrence of breast cancer patients (p < 0.05, Figures 2D,E). Further, we counted the expression of DEPDC1 in triple-negative breast cancer (TNBC) which was a highly malignant breast cancer, and observed that its expression in TNBC even higher (p < 0.0001, Figure 2F). Therefore, the above results suggested that DEPDC1 was associated with breast cancer progression and might have prognostic significance for breast cancer patients.

### High Expression of DEPDC1 Is Correlated With Poor Clinical Outcome of Breast Cancer Patients

**Figure 3:**
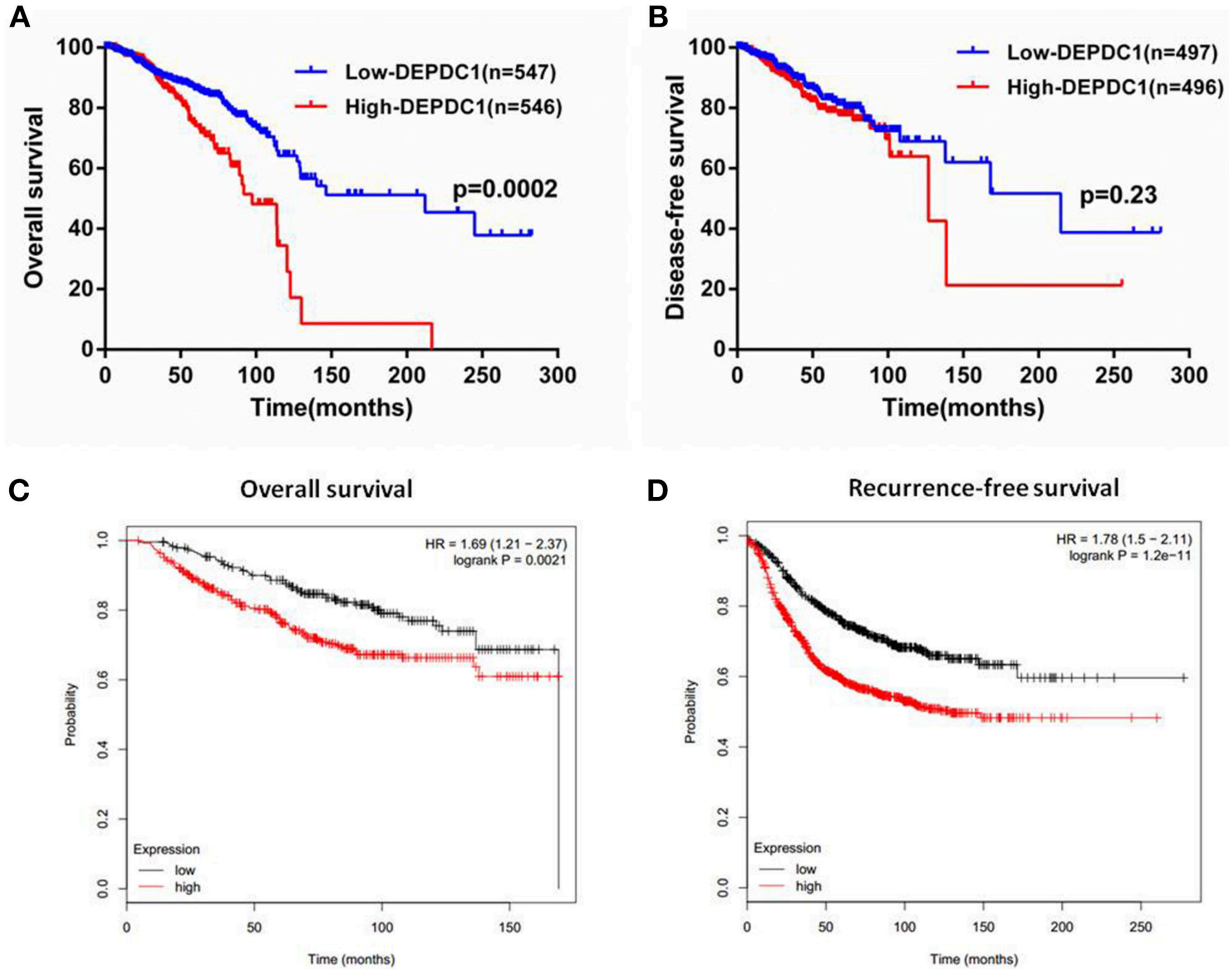
High level of DEPDC1 mRNA predicted poor prognosis of breast cancer patients. Patients were separated into high- or low-DEPDC1 expression groups using median as the cutoff. **(A)** High DEPDC1 expression level was significantly associated with shorter OS time of breast cancer patients (*p* < 0.001). **(B)** Kaplan–Meier (KM) curves revealed a shorter DFS time with high DEPDC1 expression level though there was no significant difference (*p* > 0.05). **(C,D)** Association between DEPDC1 expression and overall survival (**(C)**, *p* < 0.05) and Recurrence-free survival (**(D)**, *p* < 0.05) was performed with the online Kaplan–Meier survival analysis (KMplot, http://kmplot.com/analysis/).

To investigate whether DEPDC1 was a potentially as a prognostic marker, the overall survival (OS), and disease-free survival (DFS) time of breast cancer patients from the follow-up data were performed by Kaplan-Meier survival analysis. Patients from TCGA_BRCA data set were separated into “high” or “low” group based on the median value of DEPDC1mRNA. The results suggested that high DEPDC1mRNA level was significantly associated with shorter OS of breast cancer (Figure 3A), while DEPDC1 expression level had no significantly predictive value for patients during DFS (Figure 3B) owing to breast cancer did not recrudesce in so short time. Further, we analyzed the association between DEPDC1 expression and survival time of breast cancer patients using an online Kaplan–Meier survival analysis tool (KMplot, http://kmplot.com/analysis/) and the patients were split by auto select best cutoff. The Kaplan-Meier analyses showed that DEPDC1 low expression group had longer OS and DFS than DEPDC1 high expression group in terms of survival duration, particularly in DFS (Figures 3C,D). Multiple databases jointly certificated that DEPDC1 could be a prognostic marker for breast cancer.

### DEPDC1 Promotes Cells Proliferation and Drives Cell Cycle Transition in Breast Cancer Cells

**Figure 4:**
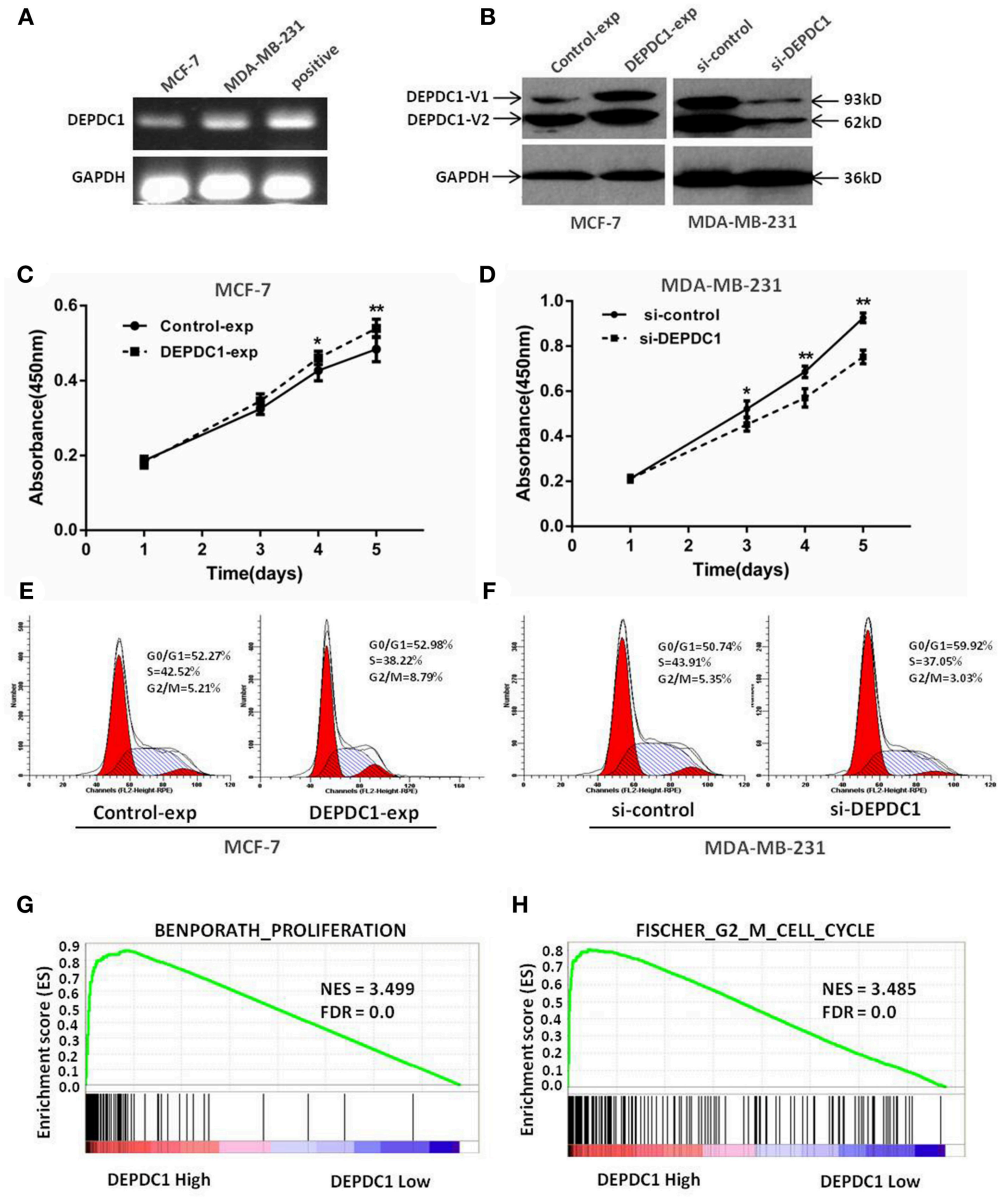
DEPDC1 promoted cells proliferation and drived cell cycle transition in breast cancer cells. **(A)** Expression of DEPDC1 in MCF-7 and MDA-MB-231 cells was determined using RT-PCR. **(B)** DEPDC1 was stably overexpressed in MCF-7 cells and silenced in MDA-MB-231 cells by transfection and selection. DEPDC1 expression was confirmed by western blot analysis. **(C,D)** Viability of breast cancer cells after being processed was evaluated using CCK-8 assay, which suggested DEPDC1 promoted the proliferation of breast cancer cells. **(E,F)** Cycle distribution analysis of breast cancer cells with altered DEPDC1 expression were performed by flow cytometry. **(G)** Enrichment plots of gene expression signatures for cell proliferation and cell cycle **(H)** were analyzed according to DEPDC1 mRNA expression level by GSEA of TCGA_BRCA dataset. False discovery rate (FDR) gives the estimated probability that a gene set with a given normalized ES (NES) represents a false-positive finding; FDR <0.25 is a widely accepted cutoff for the identification of biologically significant gene sets.

We next assessed the biological functions of DEPDC1 in breast cancer by performing the methods of overexpression and deletion in MCF-7 and MDA-MB-231 cell lines. The RT-PCR results demonstrated that DEPDC1 expression was low in MCF-7 cells and high in MDA-MB-231cells (Figure 4A). The expression of DEPDC1 was significantly overexpressed in MCF-7 cells and depleted in MDA-MB-231cells both at protein level (Figure 4B). The CCK-8 assay showed that DEPDC1 overexpression increased the proliferation while DEPDC1 depletion inhibited the proliferation rate in breast cancer cells (Figures 4C,D). Subsequently, flow cytometry analysis revealed that DEPDC1 significantly increased the portion of G2/M phase, but reduced the portion of M phase in MCF-7 cells (Figure 4E). In contrast, the silence of DEPDC1 resulted in cell cycle arrest in MDA-MB-231cells (Figure 4F). We further used GSEA to confirm the correlation between DEPDC1 expression level and cells proliferation in breast cancer. The patients from TCGA_BRCA dataset were divided into DEPDC1 high- and low- expression groups based on the median value of DEPDC1 mRNA, and the relationship of DEPDC1 expression level with cell proliferation/G2_M cell cycle was analyzed with GSEA. As shown in Figures 4G,H, the gene signatures of both cell proliferation and G2_M cell cycle were highly enriched in patients with DEPDC1 high expression. All these results suggested that a positive correlation between DEPDC1 expression level and cell proliferation or cell cycle transition in breast cancer.

### DEPDC1 Improves the Migration and Invasion of Breast Cancer Cells

**Figure 5:**
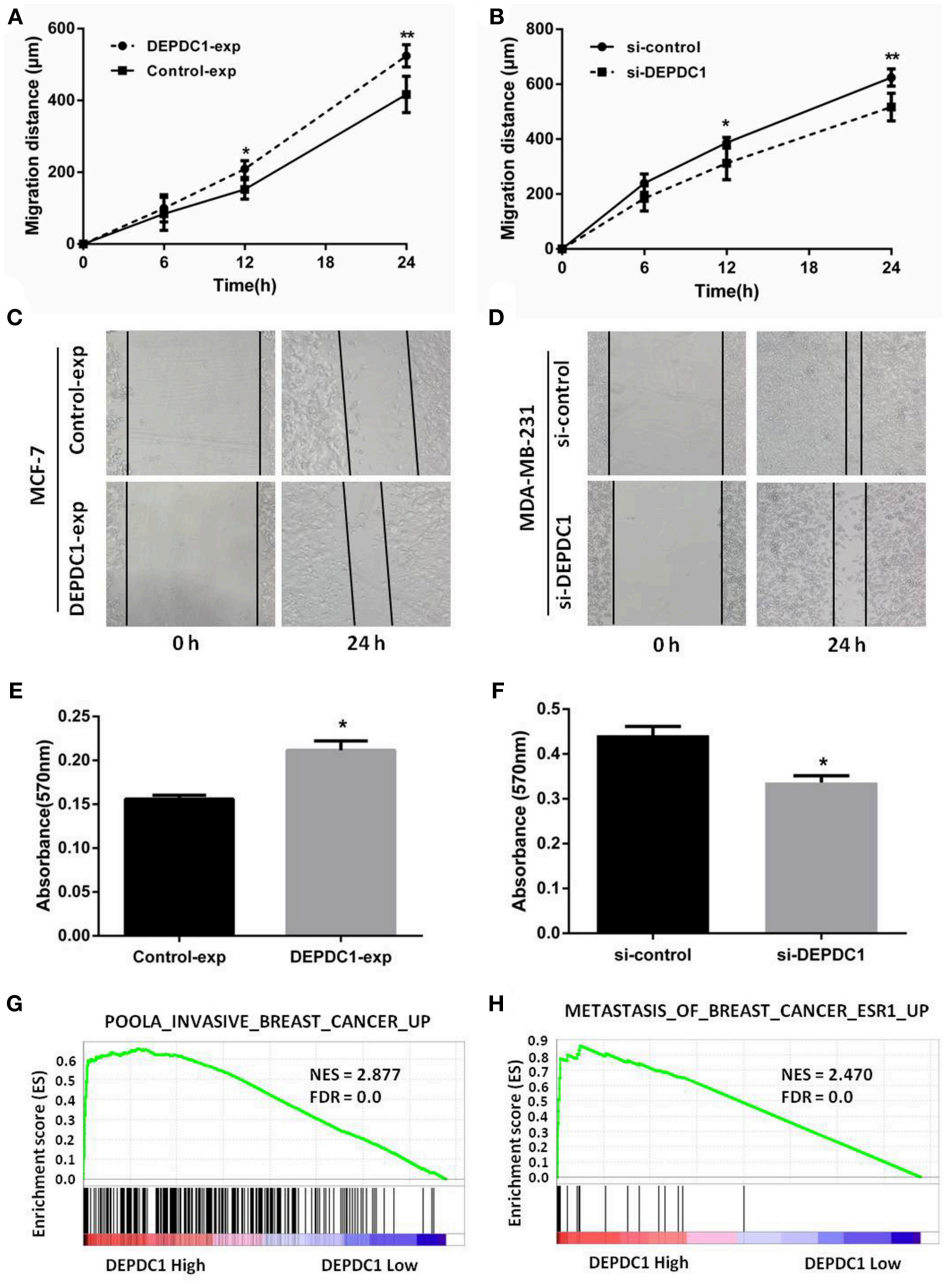
DEPDC1 improved the migration and invasion of breast cancer cells *in vitro* assay. **(A)** overexpression of DEPDC1 promoted MCF-7 cells migration distance. **(B)** Decreased migration rate was determined in DEPDC1-deletion MDA-MB-231 cells. **(C,D)** The representative images of the migration assays for MCF-7 and MDA-MB-231 cells at 0 and 24 h were presented. **(E,F)** Cells were subjected to invasion assay after altering DEPDC1 expression 48 h using transwell assay. **(G)** GSEA showed that DEPDC1 high expression was positively correlated with invasive breast cancer. **(H)** The gene signatures of breast cancer metastasis were highly enriched in patients with DEPDC1 high expression. *p < 0.05, **p < 0.01.

Migration and invasion affected the metastasis of cancer cells. Wound healing assay revealed that DEPDC1 overexpression significantly facilitated the migration of MCF-7 cells compared with control group, while knockdown reduced the migration rate in MDA-MB-231cells monolayer (Figures 5A,B). Figures 5C,D showed the representative images of the migration assays for MCF-7 and MDA-MB-231 cells at 0 and 24 h. The transwell assay demonstrated that, compared with the control groups, the overexpression of DEPDC1 in MCF-7 cells significantly stimulated the invasion ability of breast cancer cells (Figure 5E). In contrast, the deletion of DEPDC1 had opposite effects on MDA-MB-231 cells (Figure 5F). Also we explored the relationship of DEPDC1 expression level with invasion and metastasis using GSEA methods. We found significant enrichment of invasion and metastasis gene modules in breast cancer patients with high DEPDC1 expression (Figures 5G,H). In summary, this section signified that the expression of DEPDC1 was positively correlated with the migration and invasion in breast cancer.

### DEPDC1actives PI3K/AKT/mTOR Signaling in Breast Cancer

**Figure 6:**
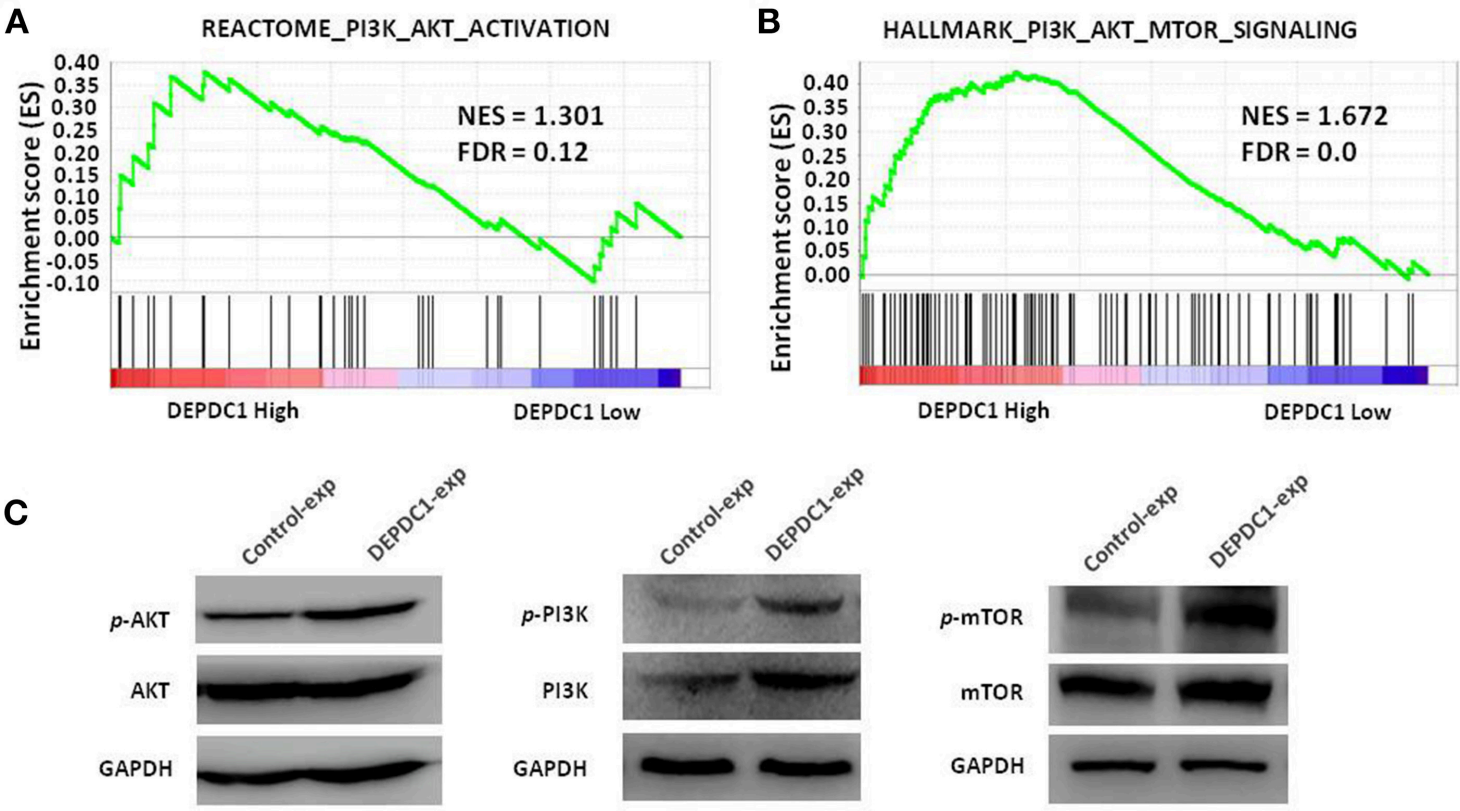
DEPDC1 activated PI3K/AKT/mTOR signaling pathway. **(A)** GSEA showed that DEPDC1 level was positively associated with PI3K_AKT_ACTIVATION gene set in breast cancer. Samples from TCGA_BRCA were divided into low- and high- DEPDC1 expression groups. **(B)** Gene signatures of PI3K/AKT/mTOR signaling activation were enriched in patients with DEPDC1-higher expression group. **(C)** The phosphorylation of PI3K p85 (Tyr458), AKT (Ser473) and mTOR (Ser2448) were activated when DEPDC1 were overexpressed in MCF-7. NES, normalized enrichment score; FDR, false discovery rate; FDR < 0.25 is a widely accepted cutoff for the identification of biologically significant gene sets.

To investigate the signaling pathway of DEPDC1 participated in, we examined classic signaling pathways associated with tumorigenesis such as ERK, NF-κB, PI3K/AKT/mTOR, and E2F using GSEA. Strikingly, DEPDC1 was only related to PI3K/AKT/mTOR signaling pathway and key genes involved in PI3K/AKT/mTOR signaling activation were enriched in patients with higher DEPDC1 expression (Figures 6A,B). In addition, the PI3K/AKT/mTOR signaling pathway acts a crucial role in multiple cellular processes that contribute to the malignant phenotype of breast cancer (22, 23). Therefore, we further confirmed the effect of DEPDC1on PI3K/Akt/mTOR signal pathway using western blot analysis. The results showed that DEPDC1 overexpression significantly promoted the phosphorylation of PI3K p85 (Tyr458), AKT (Ser473), and mTOR (Ser2448) in MCF-7 cells (Figure 6C). These findings suggested that in breast cancer, the expression of DEPDC1 is positively associated with PI3K/AKT/mTOR activation.

## Discussion

DEPDC1 is highly expressed in various kinds of cancers, like bladder cancer, hepatocellular carcinoma, lung cancer, and nasopharyngeal carcinoma (18, 24–27). Its abnormal expression drives the progression of carcinogenesis and the malignant behavior of cancer cells (12, 28). In spite of DEPDC1 level was upregulated in human breast cancer tissues specimens and mouse model with ductal carcinoma *in situ* (9, 19, 20), less is known about its biological functions and clinical significance in breast cancer. In the present study, we found DEPDC1 was highly expressed in breast cancer tissues. Its expression was significantly associated with tumor malignancy and advanced stage, and the high DEPDC1 level was obviously correlated with poor clinical outcome. The *in vitro* assays demonstrated that DEPDC1 promoted the proliferation, migration, and invasion, and involved in PI3K/AKT/mTOR signaling in breast cancer cells.

Currently, DEPDC1 promoting cancer development was gradually discovered and had been widely considered as a putative oncogene. The role of DEPDC1 in cancers was mainly found in bladder cancer (8, 10, 24, 29, 30). It was reported that DEPDC1 was significantly increased in bladder carcinoma and necessary for the proliferation of cancer cells (8). Moreover, DEPDC1 repressed the transcription of A20 through interacting with ZNF224, leading to activate NF-κB pathway in bladder cancer (10). In breast cancer, DEPDC1 was one of the most upregulated genes via analyzing microarray data (19). The open-access TCGA data carries out gene expression for breast cancer and provides a great resource for investigators to explore this area and to identify new methods for cancer diagnosis, treatment and prevention (31). In this study, we analyzed the transcriptional profiles of DEPDC1 in TCGA_BRCA and GEO dataset. The results showed that DEPDC1 mRNA and protein expression levels were dramatically enhanced in breast cancer tissues (Figure 1), and a step-wise increase toward tumor stage, tumor size, and distant metastasis was observed (Figures 2A–E). TNBC as a particular subtype of breast cancer, characterized with aggressive biological behavior and the lack of molecular targets for therapeutic intervention (32). As shown in Figure 2F, DEPDC1 was higher expressed in TNBC group compared with non-TNBC group. Additionally, several studies revealed that the higher expression of DEPDC1 was significantly correlated with poorer survival of patients in hepatocellular carcinoma and multiple myeloma, indicating that DEPDC1 might be a new diagnostic marker (11, 14). Consistent with these reports, the upregulation of DEPDC1 in breast cancer was notably associated with patients with shorter survival time (Figure 3).

The dysregulation of gene expression caused malignant phenotype, including hyperproliferation, hypermigration, and invasion (33). In nasopharyngeal carcinoma, silence of DEPDC1 resulted in significant reduction of proliferation, migration, invasion, and delay in cell cycle progression (18). Another reported demonstrated that DEPDC1 inhibited the proliferation of A549 cell by suppressing cells apoptosis (27). Consistently, DEPDC1 overexpression improved the growth, migration, invasion ability, and drove G1 to S phase cell cycle transition in breast cancer cells, and the deletion of DEPDC1 suppressed these malignant phenotypes (Figures 4C–F, Figures 5A–F). Moreover, DEPDC1 as a novel upregulated gene, involving in proliferation, cell cycle, invasion, and metastasis in breast cancer was disclosed by GSEA from TCGA_BRCA data set (Figures 4G,H, 5G,H). Taken together, these data represented that DEPDC1 accelerated proliferation as well as movement of malignant breast cancer cells.

Several studies manifested that the increased level of DEPDC1 might exert influence through multiple different signaling pathways. Previous studies showed that DEPDC1 inhibited the transcription of A20 through NF-κB pathway in bladder cancer, hepatic carcinoma, lung cancer, and nasopharyngeal carcinoma, which acted a key role in cell proliferation, tumorigenesis and metastasis (10, 15, 18, 26, 27, 34). The sustaining division of cancer cells primarily involved the activation of E2F signaling (35). In prostate cancer, DEPDC1 facilitated cell proliferation and tumor growth via activating E2Fsignaling pathway (12). In this study, we explored the relationship of DEPDC1 level with NF-κB and E2F signaling pathway using GSEA, and found that these two signaling gene set were no associated with DEPDC1 level in breast cancer. The activation of PI3K/AKT/mTOR signaling pathway is closely connected with clinical characteristics and poor prognosis in breast cancer (36). Intriguingly, PI3K/AKT/mTOR signaling was distinctly activated in breast cancer with high DEPDC1 expression (Figures 6A,B). The present *in vitro* data illustrated that DEPDC1 stimulated the phosphorylation of AKT, PI3K, and mTOR in MCF-7 cells (Figure 6C). This result suggested DEPDC1 might affect the malignant phenotype via activating PI3K/AKT/mTOR signaling in breast cancer. Certainly, more thorough research works are required needed to be performed for a clear conclusion.

In summary, these findings showed that DEPDC1 upregulation was associated with tumor development and poorer clinical outcomes of patients with breast cancer. *In vitro* cell function data indicated that DEPDC1 served as an oncogene in breast cells. Further, the present study provided a novel regulatory mechanism of DEPDC1 in breast cancer by strengthening PI3K/AKT/mTOR signaling to deteriorate phenotypes. Our findings provide novel thought to improve current understanding of the pathogenesis of breast cancer.

## Author Contributions

HZ designed the overall idea of this study and completed most of the experiments. MY and LS assisted in downloading the GEO and TCGA data set. BG completed the immunohistochemistry experiments. BZ did some statistics. JC provided the cell lines and slides. CH guided and supervised this study and ZG provided the clinical samples and reviewed the manuscript.

### Conflict of Interest Statement

The authors declare that the research was conducted in the absence of any commercial or financial relationships that could be construed as a potential conflict of interest.
