# Supplementary material for: High Expression of DEPDC1 Promotes Malignant Phenotypes of Breast Cancer Cells and Predicts Poor Prognosis in Patients With Breast Cancer
Source: Front Oncol. 2019 Apr 12;9:262. doi: 10.3389/fonc.2019.00262 (PMC6473048; doi:10.3389/fonc.2019.00262)
Supplement: Supplementary file 1 [file Table_1.DOCX]

**Supplementary Table 1: Oligonucleotides used in the present study**

**Primers used for RT-PCR analysis**

| **Gene name** |  | **Primer sequence** |  |
| --- | --- | --- | --- |
| DEPDC1 |  | F : 5'-ACGAAGGTATCCAGAATTG-3’  R: 5'-AGATAATACCCAGTGAGGGA-3'  F: 5'-GGCTGCTTTTAACTCTGGTA-3'，  R: 5'-GACTGTGGTCATGAGTCCTT-3' | |
| GAPDH |  |  |  |

**siRNAs**

| **Name** | **Sequences** |
| --- | --- |
|  |  |
| DEPDC1 siRNA | Sense:5'-TCAGGAGTTTGATGAGCAACTCGAG TTGCTCATCAAACTCCTGAGC -3' |
| Negative control siRNA | Sense: 5'- TTCTCCGAACGTGTCACGT-3' |
